# Influence of the Belgian Coast on Well-Being During the COVID-19 Pandemic

**DOI:** 10.5334/pb.1050

**Published:** 2021-09-20

**Authors:** Marine I. Severin, Michiel B. Vandegehuchte, Alexander Hooyberg, Ann Buysse, Filip Raes, Gert Everaert

**Affiliations:** 1 Flanders Marine Institute (VLIZ), Ostend, Belgium; 2 Department of Experimental Clinical and Health Psychology, Ghent University, Belgium; 3 Centre for the Psychology of Learning and Experimental Psychopathology, KU Leuven, Belgium

**Keywords:** coast, well-being, COVID-19, awe, nostalgia

## Abstract

There is increasing evidence that blue spaces, particularly coastal environments, are beneficial for well-being. During the first-wave lockdown of the COVID-19 pandemic in Belgium, access to the coast was restricted due to restraint in circulation. Making use of this unique opportunity, this study investigated whether access and visits to the coast were positively associated with well-being by using a quasi-experimental design. The emotions of awe and nostalgia were studied as potential mediators between coastal visits and well-being. A total of 687 Flemish adults took part in an online survey that was launched end of April until beginning of June 2020. After controlling for covariates, results showed that access to the coast, but not visit frequency, was positively associated with well-being. More specifically, coastal residents reported less boredom and worry, and more happiness than inland residents. Awe and nostalgia were not significantly associated with coastal visits, but awe was negatively correlated with boredom. The study suggests a potential buffer effect of residential proximity to the coast against negative psychological consequences of the COVID-19 pandemic, supporting the notion that the coast has a positive impact on well-being.

The COVID-19 pandemic that arose in December 2019 has led to many restrictions for people’s daily activities. One of these restrictions was that during Belgium’s national lockdown between March 18^th^ and June 8^th^ 2020, a large proportion of its population no longer had access to the coast. This situation led to a chance to use a quasi-experimental design to assess the effect of coastal environments on well-being.

The relationship between coastal environments and well-being has recently been the subject of several studies. Coastal environments are part of what are called *blue spaces*, which are defined as “outdoor environments, either natural or manmade, that prominently feature water and are accessible to humans either proximally (being in, on, or near water) or distally/ virtually (being able to see, hear or otherwise sense water)” (Grellier et al., 2017, p. 3). In a systematic review, Gascon et al. (2017) reveal a positive association between greater exposure to blue spaces and benefits to mental health and well-being. Some studies found that the positive effects of blue spaces might even be stronger than those of green spaces such as parks, forests, and fields (White et al., 2013b).

Investigating the relationship between blue spaces and well-being is becoming increasingly valuable in today’s context. On the one hand, researchers discern a growing trend in human disconnection from nature (Kesebir & Kesebir, 2017). On the other hand, further deterioration in mental health is becoming apparent with regards to the COVID-19 pandemic, as recently demonstrated by Vindegaard and Benros (2020). Understanding how and why blue spaces benefit mental health could help us optimize the use of these spaces to potentially build resilience in mental health, especially in times of crisis.

## Well-being

Well-being is a complex concept that can be defined according to two different perspectives. The first perspective, hedonism (also referred to as subjective well-being), defines well-being as a state of positive affect and absence of negative affect (Kahneman et al., 1999). Indicators such as life satisfaction and happiness are typically used to measure hedonic well-being. The second perspective, eudaimonism, views well-being as more than just happiness but as the result of living in accordance with one’s true self (Waterman, 1993). Eudaimonic well-being can be conceptualized with the six dimensions posited by Ryff and Keyes (1995) namely, autonomy, environmental mastery, personal growth, positive relationships, purpose in life, and self-acceptance. Both perspectives are taken into account in the present study.

Studying how to preserve and promote well-being is fundamental due to its impact on health and society (Maccagnan et al., 2019). Nonetheless, research on the factors that affect well-being has been largely centered around individual and social parameters such as personality traits (Sirgy, 2021) and social support (Taylor, 2011). There is a lack of investigation on the role of the environment on well-being (Schleicher et al., 2018). Environmental factors are however central to the development of well-being as displayed, for example, by the negative effect of urbanization on well-being. For instance, studies show a higher prevalence of mood and anxiety disorders (Peen et al., 2010) and a lower life satisfaction (Sørensen, 2014) in urban residents compared to rural residents. Considering that 68% of the world’s population is projected to live in an urban area by 2050 (Population Division, 2019), it is becoming crucial to investigate the role of the environment on well-being and provide solutions to promote a healthy relationship between the two. One important aspect that is being increasingly looked into is the benefit of nature, and, more specifically coastal environments, on well-being.

## The Coast and Well-being

Evidence that coastal environments have a positive impact on mental health is being progressively gathered. A systematic review of therapeutic blue space interventions indicated a positive association with mental health indicators such as increased psychological restoration, of which 50% of the studies focused on the coast (Britton et al., 2020). Studies in England (Wheeler et al., 2012; Garrett et al., 2019a; see also White et al., 2013a for longitudinal data) and Belgium (Hooyberg et al., 2020) found that living near the coast is beneficial, as residential proximity to the coast was linked with a significantly better general health and mental health compared to residents living inland. Furthermore, visits to the coast have also been associated with improved well-being in studies done in Spain (Vert et al., 2020) and Hong Kong (Garrett et al., 2019b). Finally, higher coastal visibility from one’s home was related to lower psychological distress in a study implemented in New Zealand (Nutsford et al., 2016). There is therefore a strong basis for stating that the coast has a beneficial effect on well-being.

## Mechanisms

Despite the increasing evidence of a positive effect of the coast on well-being, the suggested explanations or mechanisms of this effect are not yet fully investigated. Physical activity has been advocated as a potential mechanism, as coastal proximity is positively associated with an increased likelihood of physical activity thereby possibly explaining its benefits on well-being (White et al., 2014). Moreover, in the UK, an initiative called “The Blue Gym” was launched in order to promote the physical and mental health benefits from doing exercise along the coast (Depledge & Bird, 2009). Other studies manifest that the multisensory stimulation of the coast can explain its restoration effects, such as the sounds of the breaking waves or the reflection of light on the water as it creates fractal patterns (Bell et al., 2015; White et al., 2010).

The emotional experience that the coast brings has been proposed as yet another potential mechanism (White et al., 2020). Amongst the many emotions that the coast potentially induces are the emotions of awe and nostalgia (Willis, 2015; Pearce et al., 2017). Awe is triggered by anything that is experienced as being larger than the self, and leads to a need for accommodation to assimilate this experience (Keltner & Haidt, 2003). Qualitative interviews conducted with visitors at a seaside resort demonstrate that the coast can provoke awe-inspiring experiences, due to its grandeur and sublimity (Jarratt & Sharpley, 2017). Participants also mentioned that the coast generated feelings of nostalgia (Jarratt & Gammon, 2016), defined as “a wistful or excessively sentimental yearning for return to or of some past period or irrecoverable condition” (Merriam-Webster, n.d.). The emotion was described to arise from a reconnection with childhood memories created at the coast and the coast’s sharp contrast with one’s often fast-paced daily life (Jarratt & Gammon, 2016).

Both awe and nostalgia seem to be linked to the aspect of timelessness of the coast through its perceived unchanging nature and broad horizon. This aspect of timelessness can perhaps be an antecedent to a perception of expanded time availability which has been shown to mediate the relationship between awe and momentary life satisfaction (Rudd et al., 2012). Anderson et al. (2018) also found awe to positively mediate the effect of nature experiences on daily life satisfaction and improved well-being at follow-up. In parallel, Hepper et al. (2021) demonstrate that nostalgia helps maintain well-being in the face of limited time perspectives and is an important resource to cope with distress (Sedikides & Wildschut, 2016). Nostalgia and awe have both also been associated with an enhancement in meaning in life: awe by placing the self within a bigger picture and gaining perspective on life (Danvers et al., 2016) and nostalgia by boosting social connectedness (Routledge et al., 2012). Meaning in life is seen as an essential contributor to well-being (Steger, 2009). Taken together, there is a clear rationale to investigate whether coast-induced awe and/or nostalgia positively influence well-being.

## The COVID-19 Pandemic

The COVID-19 pandemic presents a deep contrast with normal living conditions and has been a global source of stress and anxiety. A systematic review of studies from various countries by Vindegaard and Benros (2020) demonstrates a decrease in psychological well-being and an increase in scores of depression and anxiety in the general public, compared to before COVID-19. Similar results have been found for the Belgian population, with the majority of the population also suffering from a lack of good quality sleep (Charafeddine et al., 2020).

In order to slow down the further spread of the coronavirus, many countries resorted to extreme measures such as restricting circulation. In Belgium, the government issued a state “lockdown” on March 18 2020, meaning that all citizens were asked to stay at home and could only go outside for essential trips or outdoor physical activity not far from one’s own home. Circulation between cities was not allowed. Consequently, those who did not live close to the coast no longer had access to coastal environments, until June 8 2020.

It has been shown that quarantine can lead to negative psychological effects such as post-traumatic stress symptoms, with some stressors including frustration, boredom, fears, and inadequate information (Brooks et al., 2020). Faced with the strong psychological impact of the coronavirus, we wanted to investigate whether being exposed to the coast could potentially buffer this impact.

## The Present Study

Our study aimed to evaluate whether access and visits to the coast would be associated with higher well-being during the first-wave lockdown, as well as suggest a possible pathway to explain this association. The COVID-19 pandemic provided an opportunity to investigate the relationship between the coast and well-being within a quasi-experimental design. Using an online survey, three hypotheses were tested. We first examined whether those who had access to the coast experienced higher levels of well-being than those who did not have access (H1). We then evaluated whether more frequent visits to the coast were positively associated with well-being (H2). Finally, we wanted to test whether awe and/or nostalgia were mediators in the relationship between coastal visits and well-being, in the condition that the pre-requisites of mediation were fulfilled (H3).

## Method

### Participants

We launched an online survey, available through the platform Limesurvey, on April 22^nd^ until June 8^th^. A total number of 687 participants took part in the survey. The percentage of participants who identified as women was about 68.9% (*n* = 473), 30.9% of the participants identified as men (*n* = 212), and 0.3% of the participants identified as “other” (*n* = 2). The age range was from 19 to 81 years old (*M* = 43.29, *SD* = 15.37), with 65.8% being older than 40 years old. Almost half (46%) were educated on a university level and 45.4% declared working from home due to the coronavirus pandemic. The majority of participants lived in Flanders, the Dutch speaking part of Belgium (97.7%). Of the total sample, 44.3% lived in the westernmost province of Flanders, i.e. West Flanders, and 24.2% lived up to 5 km away from the coast. In consideration of these numbers, it is important to mention that our sample is not representative of the entire Dutch-speaking population of Belgium.

### Measures

The online survey consisted of a series of questions aiming to assess the effect of the coast on well-being during the first-wave lockdown. The survey was presented in Dutch. In the questionnaire, we evaluated access to the coast and visit frequency (predictor variables), frequency of emotions during visits to preferred environment (mediator), well-being (outcome variable), and various control variables.

#### Access to the coast and visit frequency

To measure access to the coast, participants were asked to report their postal code. Visit frequency was assessed by asking participants how often they visited the following environments during the lockdown: coast (e.g. beach, dunes, dike), natural green spaces (e.g. city parks, forests), countryside (e.g. agricultural areas), urban or urbanized spaces (e.g. village and city centers), and their own balcony, terrace, or garden. Response options were *never, less than once a week, once a week, more than once a week*, and *every day*.

#### Frequency of emotions during visits to preferred environment

Before asking about their emotions, participants needed to indicate which environment they preferred to visit during the lockdown. They then could report the frequency of the following emotions when they visit that particular environment: satisfied, angry, anxious, in awe, grateful, sad, nostalgic, proud, amused, relaxed. Response options were: *never, seldom, sometimes, often*, and *always*.

#### Well-being

Three dimensions of well-being were assessed. The first, general well-being, was measured by the Dutch version of the Short Warwick Edinburgh Mental Well-being Scale (SWEMWBS; Stewart-Brown et al., 2009; Ikink et al., 2012). The SWEMWBS was developed by the Universities of Warwick, Edinburgh and Leeds in conjunction with NHS Health Scotland. Several items in this seven-item scale represent aspects of eudaimonic well-being such as agency, social connection, and clarity of thoughts, while other items represent aspects of hedonic well-being such as positive affect. Subjects were asked to choose the best option that suits them according to certain thoughts and feelings experienced over the past two weeks, going from *never* to *always*. In this sample, Cronbach’s alpha for the SWEMWBS was .81. The second dimension, negative experiential well-being, was represented by items on worry, stress, and boredom. These items were evaluated on a seven-point Likert scale, with responses on the lower end representing an experience of *less than usual* and on the higher end *more than usual*, over the past two weeks. For the third dimension, positive experiential well-being, participants were asked to rate their current happiness on a 10-point scale, going from *extremely unhappy* to *extremely happy*.

#### Control variables

As this was a quasi-experimental study, several influential variables for well-being needed to be controlled for. Participants were asked to report their age, gender, level of education, and presence or absence of a mental illness. Level of education was used as an indicator of socio-economic status as it particularly depicts effects on health and lifestyle (Shavers, 2007). Participants also indicated the extent to which the quality of their sleep and frequency of physical activity had changed since the beginning of the lockdown, using a scale from 1 (*I sleep much worse*) to 5 (*I sleep much better*) for sleep quality and 1 (*a lot less*) to 5 (*much more*) for physical activity. Variables that were important when considering the effect of the lockdown such as household composition (e.g. “alone with children”, “couple with no children”) and change in work situation (e.g. “working from home”, “temporarily unemployed”) were included.

### Procedure

Participants were recruited by releasing a message to the press via the Flanders Marine Institute website on April 22^nd^ (VLIZ, 2020). Advertisement of the survey was also spread through social media networks and via the newsletter of the province of West Flanders. The ad contained the link to the survey, its estimated duration time, and the aim of the study, namely, to examine how outdoor visits influence our emotions and our health, given the current circumstances related to COVID-19.

Upon opening the link to the questionnaire, subjects were presented with a short introduction and an informed consent form. After finishing the survey, participants were thanked and were informed about the current measures that help protect oneself and others of the coronavirus, official government information sources, and contact details of a free telephone and chat support service that offers a listening ear for anyone that needs it. Participants were also offered the chance to send an email to the researchers in order to receive a debriefing or to ask any questions they had.

### Statistical Analysis

To determine the effect of access to the coast on well-being, i.e. the first hypothesis, participants were assigned to one of two groups based on their postal code. Only those living in municipalities which border the Belgian shoreline (“coastal residents”; *n* = 168) had access to the coast during the lockdown due to the restriction of circulation. Respondents living in other municipalities (“inland residents”; *n* = 519) did not have access. For the second hypothesis, coastal residents were divided into three groups with different visit frequencies. The first group visited the coast once a week or less (*n* = 43), the second group visited the coast more than once a week (*n* = 67), and the last group visited the coast every day (*n* = 58). Considering the high intercorrelations between the indicators of well-being, multivariate analyses of covariance (MANCOVA) were used to test the two hypotheses. As follow-up, univariate analyses of covariance (ANCOVA) were employed to assess the effect on each outcome variable. Covariates that were associated with one or several outcome variables were included in the models. Multivariate outliers were identified by calculating the Mahalanobis distance and were consequently taken out of the analysis. All tests were conducted with an alpha significance level of .05.

The MANCOVAs were performed with all five indicators of well-being as outcome variables, and access to the coast (H1) and visit frequency (H2) as predictor variables. For the first model (H1), gender, mental illness, household composition, sleep quality, and physical activity were included as covariates. Five outliers were taken out and no serious violations were detected, except for a significant interaction between access to the coast and the covariate household composition, *F* (20, 2632) = 1.80, *p* = .016, violating the assumption of homogeneity of regression slopes. This interaction term was therefore included in the model. For the second model (H2), household composition and sleep quality were included as covariates. Four outliers were removed and the assumptions were met. To test the third hypothesis (H3), a one-way ANOVA was used to assess the relationship between the preferred environment of the coastal residents and the frequency of awe and nostalgia felt in that environment. Then, Pearson correlations were conducted to examine the associations between awe and nostalgia with the indicators of well-being, for coastal residents whose preferred environment was the coast.

## Results

### Coastal Residents versus Inland Residents

Results showed higher well-being for coastal residents compared to inland residents during the lockdown, while controlling for gender, mental illness, household composition, sleep quality, physical activity, and the interaction between access to the coast and household composition. Based on Pillai’s Trace, the MANCOVA indicated a significant association between access to the coast and the combined indicators of well-being, *V* = .024, *F* (5, 655) = 3.23, *p* = .007, with a small effect size (η^2^ = .024), as shown in ***Table 1***. Follow-up univariate ANCOVAs on the outcome variables demonstrated differences for boredom, *F* (1, 659) = 4.91, *p* = .027, η^2^ = .007, worry, *F* (1, 659) = 9.15, *p* = .003, η^2^ = .014 and happiness, *F* (1, 659) = 6.89, *p* = .009, η^2^ = .010. Coastal residents reported less boredom and worry and more happiness during the lockdown than inland residents. Indeed, the estimated marginal means (which are adjusted for the covariates) show a relative difference of –9.1% for both boredom and worry, and a relative difference of +5.5% for happiness, for coastal residents compared to inland residents (***Table 2***). The covariates of age and level of education, which are typically considered as influential to well-being, were not included in the final model as they were not statistically relevant in this context. Note that including them did not meaningfully change our results.

**Table 1:** Multivariate and Univariate Analyses of Covariance for Well-being as a Function of Access to the Coast, With Covariates.


	MULTIVARIATE		UNIVARIATE									

			BOREDOM		WORRY		STRESS		HAPPINESS		GENERAL WELL-BEING	

VARIABLE	*F*	η^2^	*F*	η^2^	*F*	η^2^	*F*	η^2^	*F*	η^2^	*F*	η^2^

Access to coast	3.23**	.024	4.91*	.007	9.15**	.014	1.66	.003	6.89**	.010	.90	.001

Household composition	2.85***	.021	.66	.004	3.23*	.019	4.17**	.025	3.73**	.022	1.24	.007

Gender	2.24*	.017	1.87	.006	.59	.002	4.14*	.012	1.53	.005	1.96	.006

Mental Illness	5.00***	.037	1.16	.004	1.39	.004	9.51***	.028	22.2***	.063	9.53***	.028

Sleep quality	33.78***	.205	26.34***	.038	110.3***	.14	126.7***	.16	78.19***	.11	74.24***	.101

Physical activity	2.44*	.018	2.14	.003	8.71**	.013	6.63**	.010	4.47*	.007	.80	.001

Access to coast x Household composition	1.80*	.013	3.79**	.022	1.53	.009	.27	.002	2.22	.013	.34	.002


*Note*. *p < .05. **p < .01. ***p < .001.

**Table 2:** Estimated Marginal Means of Well-Being for Coastal and Inland Residents.


VARIABLE	GROUP	*M*	[MIN, MAX]	*SE*	95% CI

Boredom	Inland	2.92	[1, 7]	.44	[2.07, 3.78]
	
	Coastal	2.28		.52	[1.27, 3.30]

Worry	Inland	4.47	[1, 7]	.31	[3.84, 5.05]
	
	Coastal	3.83		.36	[3.11, 4.55]

Stress	Inland	3.98	[1, 7]	.32	[3.36, 4.59]
	
	Coastal	3.71		.37	[2.97, 4.44]

Happiness	Inland	5.99	[1, 10]	.32	[5.36, 6.61]
	
	Coastal	6.54		.38	[5.80, 7.28]

General well-being	Inland	21.01	[7, 35]	.76	[19.52, 22.49]
	
	Coastal	21.48		.90	[19.71, 23.24]


### Visit Frequency in Coastal Residents

Coastal residents who visited the coast daily during the lockdown did not differ in well-being from those who visited the coast more than once a week or less, while controlling for household composition and sleep quality. Based on Pillai’s Trace, visit frequency had no significant association with the indicators of well-being, *V* = .094, *F* (10, 310) = 1.54, *p* = .126, with a small effect size, η^2^ = .047 (***Table 3***). Including participants’ preferred environment as a covariate did not meaningfully change our results.

**Table 3:** Multivariate Analysis of Covariance for Well-being as a Function of Visit Frequency to the Coast, With Covariates.


VARIABLE	*F*	η^2^

Visit frequency	1.54	.047

Household composition	1.59*	.048

Sleep quality	12.37***	.287


*Note*. *p < .05. **p < .01. ***p < .001.

### Mechanisms of Awe and Nostalgia

Coastal residents who preferred to visit the coast (*n* = 89) did not experience awe nor nostalgia during their visits more frequently than those who preferred to visit natural green spaces (*n* = 28), the countryside (*n* = 6), or their own balcony, terrace, or garden (*n* = 45). Results were non-significant for both awe, *F* (3, 164) = 1.36, *p* = .256, and nostalgia, *F* (3, 164) = .85, *p* = .468, with small effect sizes (η^2^ = .024 and η^2^ = .015, respectively). No participants chose urban space as their preferred environment; therefore, it was not included in our analysis.

For coastal residents whose preferred environment was the coast, awe was found to be significantly negatively correlated with boredom, *r* = –.32, *p* = .003, but was not correlated with the other outcome variables. Nostalgia did not correlate with the well-being variables (***Table 4***). In conclusion, as there was no significant association between coastal visits and the frequency of awe and nostalgia, we could not further investigate a possible mediation of awe or nostalgia in the relationship between the coast and well-being. However, there is a negative relationship between awe felt at the coast and boredom, such that the greater the frequency of awe, the lower the experience of boredom.

**Table 4:** Intercorrelations Between Awe, Nostalgia, and Well-being Indicators for Coastal Residents with the Coast as Preferred Environment.


VARIABLE	NOSTALGIA	BOREDOM	WORRY	STRESS	HAPPINESS	GENERAL WELL-BEING

Awe	–.09	–.32**	–.15	–.13	.17	.17

Nostalgia	–	.03	–.03	–.04	.01	.02

Boredom	–	–	.34**	.36***	–.46***	–.43***

Worry	–	–	–	.71***	–.37***	–.49***

Stress	–	–	–	–	–.53***	–.61***

Happiness	–	–	–	–	–	.63***


*Note*. **p < .01. ***p < .001.

## Discussion

The present study demonstrates that those who had access to the coast during the first-wave lockdown in Belgium self-reported higher well-being than those who did not have access. Our findings are in accordance with those of Hooyberg et al. (2020) who found that living in proximity to the Belgian coast (i.e. <5 km from the coast) was linked with a better self-reported general health. Additionally, in the context of COVID-19, results mirror those of a recent study indicating that lockdown severity worldwide negatively affected mental health, but that contact with nature buffered this effect (Pouso et al., 2021). More specifically, depression and anxiety increased with greater lockdown severity, but less so if individuals had access to private outdoor spaces and if their home views included natural elements. Our study produces similar findings because, despite the lockdown, coastal residents were less likely to report an increase in boredom and worry, and lower happiness, than inland residents. Hence, having access to the coast acted as a potential buffer against negative psychological consequences of COVID-19.

Despite support for the positive impact of access to the coast, more frequent visits to the coast were not significantly associated with higher well-being. An exposure-response relationship between visit frequency in nature and eudaimonic well-being was found by White et al. (2017). The authors detected significant differences with “*never visiting*”, starting from “*once/twice a month*” to “*once a week*”, “*several times a week*” and “*every day*”. Nonetheless in our study, the limited sample size of coastal residents did not allow to include meaningful categories for visiting the coast less than once a week. Another factor that might have played a role is that experiencing a lockdown could have been accompanied with fears of being outdoors, such as encountering other people and contracting the coronavirus. These fears could have potentially counteracted the benefits of visiting the coast. Finally, it could be that in comparison to those who visited the coast several times a week, those who felt the need to visit every day were more anxious or bored and their daily visits did not help alleviate these feelings.

Exposure to blue or green spaces can be conceptualized into three types: incidental, indirect, or intentional (Keniger et al., 2013). Indirect exposure involves any experience of nature without necessarily being present in nature, such as watching nature television programs, or having a home view of the coast. Incidental exposure entails encountering nature but unintentionally, such as passing by the coast while cycling to work. Intentional exposure is having the direct intention to be present in a natural space, such as visiting the coast (Keniger et al., 2013). The present study found support for a beneficial influence of residential proximity to the coast which can be considered as a combination of the three types of exposure as being proximal to the coast increases the chances of having a sea view (indirect), incidentally encountering it (incidental), and directly visiting it (intentional) (White et al., 2020). Nonetheless, benefits of intentional exposure of the coast were not supported in our study. This is in contrast to other studies that investigated the effect of nature on mental health during the COVID-19 pandemic. Both Soga et al. (2021) and Dzhambov et al. (2021) demonstrate positive effects of indirect (e.g., view of greenery from indoors) and intentional (e.g., frequency of greenspace use) exposure on mental health. However, Soga et al. (2021) do mention that the indirect effect was greater than the intentional effect. Further research should aim at assessing the differential effect of nature exposure on mental health.

Because we did not observe a significant association between coastal visits and well-being, we could not test whether the emotions of awe and/or nostalgia are possible mechanisms in the coast and well-being relationship. The relationship between coastal visits and awe and nostalgia was also non-significant. Participants who preferred to visit the coast during the lockdown did not experience awe nor nostalgia more frequently during their visits than participants who preferred to visit other environments. The reduced sample size of coastal residents limited the power of the statistical test to detect small effects (Wilson Van Voorhis & Morgan, 2007). Additionally, perhaps no difference was revealed as green spaces potentially have the capacity to trigger awe and nostalgia as well (Anderson et al., 2018). A stronger contrast might have been found if we could have compared the emotions at the coast with the emotions in urban spaces.

Interestingly, a negative correlation (*r* = –.32) was found between awe felt at the coast and boredom, such that the higher the frequency of feeling awe, the lower the experience of boredom. Although awe triggered by nature has been found to improve mood (Joye & Bolderdijk, 2015) and to enhance life satisfaction (Anderson et al., 2018), it has yet to be linked with a reduction in boredom. Nonetheless, boredom is shown to be associated with a lack of meaning in life (Fahlman et al., 2009), while positive awe experiences have been shown to increase meaning in life through happiness (Rivera et al., 2020). It could be that awe serves as a protective factor against boredom through its enhancement of a sense of meaning in life. In regards to nostalgia, correlations with indicators of well-being were non-significant, although studies have shown nostalgia to be a buffer against stress and boredom (Sedikides & Wildschut, 2016), and to generate positive affect (Wildschut et al., 2006). Further research is needed to investigate how strongly the coast induces awe and nostalgia and whether this explains the well-being benefits perceived at the coast.

Despite the study’s advantage of following a quasi-experimental design, certain limitations are also present. One of these limitations is the sample’s lack of diverseness, undermining the generalizability of the study. This is a common issue in internet-based survey research (Brenner, 2002). As aforementioned, the majority of the participants were women, older than 40 years old, educated on a university level, and had a stable working condition during the lockdown. A more diverse sample in terms of socioeconomic indicators could have led to different results, as these appear to moderate the effect of the coast on well-being (Garrett et al., 2019a). Furthermore, even though we have accounted for several covariates, it is still possible that other confounding variables for which we did not take into account influenced our findings. We acknowledge that experimental studies are more suitable to have control over confounding variables (Davis, 2008), therefore we suggest to experimentally compare people exposed to the coast with people exposed to green or urban spaces, to assess potential differences in well-being.

We also recognize that the effect size for access to the coast on well-being is small. However, in research on nature and well-being, it is not uncommon to find small effect sizes and these effect sizes are similar to those of other influential variables such as gender and socio-economic status (Capaldi et al., 2014; Martin et al., 2020; White et al., 2013a). Finally, the concept of perceived environmental restoration would have been valuable to include as outcome variable given its impact on mental fatigue (Berto, 2005) and its possible association with awe. In line with Attention Restoration Theory (ART), Kaplan and Kaplan (1989) posit four conditions that determine a restorative environment of which the first two could be related to awe. The first condition, fascination, refers to effortless attention towards aesthetically pleasing stimuli and awe is typically characterized as being captivated by something perceived as vast and sublime (Shiota et al., 2007). The second condition, being away, refers to a sense of distance from one’s usual thoughts and concerns and the experience of awe is accompanied with reduced self-reflective thought and increased externally directed attention (van Elk et al., 2019). We therefore suggest for future studies to look into the relationship between perceived restoration of, and awe triggered by, the coast.

### Conclusion and practical implications

The COVID-19 first-wave lockdown in Belgium was an ideal opportunity to investigate the effect of access and visits to the Belgian coast on well-being. Results demonstrate that having access to the coast during the lockdown was associated with higher well-being, but the frequency of actual visits to the coast was not. The emotions of awe and nostalgia were not more frequently experienced during coastal visits in comparison to visits to other environments. However, awe felt at the coast negatively correlated with boredom. The study confirms the importance of the coast for well-being, even in times of crisis, as it seems to have provided a buffer against psychological consequences of the COVID-19 pandemic, such as boredom and worry. The potential mechanisms of the emotions of awe and nostalgia in the relationship between the coast and well-being are not fully understood yet. However, our study suggests that awe triggered by the coast might be a protective factor against boredom.

Policy-makers should consider making use of the coast to prevent further strain on mental health for future crises. For example, in case of future lockdowns, virtual exposure of the coast could be employed to counteract possible detriments to mental health. Indeed, a study by Yeo et al. (2020) demonstrates that virtual exposure of an underwater coral reef reduced boredom and negative affect and increased positive affect and nature connectedness. Although benefits would be greater with exposure to real-life natural environments (Mayer et al., 2009), virtual exposure could be an appropriate alternative in case of strict lockdown measures or inaccessibility to nature. Furthermore, we suggest that clinical practitioners consider recommending patients to increase interactions with blue spaces, either directly or indirectly with the use of images or videos, and to focus on triggered emotions, particularily awe, as means to alleviate feelings of worry and boredom and to boost positive affect.
